# Basosquamous Basal Cell Carcinoma with Bone Marrow Metastasis

**DOI:** 10.3390/curroncol29040178

**Published:** 2022-03-23

**Authors:** Lise Mayrin Økland Thunestvedt, Lars Helgeland, Ingeborg Margrethe Bachmann, Åsa Karlsdottir, Torjan Magne Haslerud, Håkon Reikvam

**Affiliations:** 1 Department of Medicine, Haukeland University Hospital, 5021 Bergen, Norway; lise.mayrin.okland.thunestvedt@helse-bergen.no; 2 Institute of Medical Science, University of Bergen, 5007 Bergen, Norway; lars.helgeland@helse-bergen.no (L.H.); ingeborg.bachmann@uib.no (I.M.B.); 3 Department of Pathology, Haukeland University Hospital, 5021 Bergen, Norway; 4 Department of Dermatology, Haukeland University Hospital, 5021 Bergen, Norway; 5 Department of Oncology, Haukeland University Hospital, 5021 Bergen, Norway; asa.karlsdottir@helse-bergen.no; 6 Department of Radiology and Nuclear Medicine, Haukeland University Hospital, 5021 Bergen, Norway; torjan.magne.haslerud@sus.no; 7 Institute of Clinical Science, University of Bergen, 5021 Bergen, Norway

**Keywords:** basal cell carcinoma, bone marrow metastasis, nonmelanoma skin cancer, bone marrow invasion

## Abstract

Basal cell carcinoma (BCC) is the most common cancer in Caucasians. It is slow growing and rarely metastasizes. If left untreated over time, invasive growth can occur. We present a patient case with a primary BCC located in the right sub-mammary area, with extensive metastases to the skeleton and bone marrow. Histopathological examination of the tumour showed BCC with a diverse growth pattern. There were no signs of local metastases. Surgery was successfully performed. Three months post-surgery the patient developed normocytic anaemia and elevated inflammation markers. [^18^F]FDG PET/CT showed extensive FDG uptake in the entire skeleton and bone marrow. Biopsy confirmed the infiltration of BCC with similar histopathological features as the primary tumour. Prognosis of metastasized BCC is poor and, therefore, long-term follow-up of patients with risk factors is of importance.

## 1. Introduction

Basal cell carcinoma (BCC) is the most common type of skin cancer in the fair-skinned population [1]. Due to its low mortality of less than 1% [2,3], many countries do not hold statistics on the incidence of BCC. Current data suggest that the incidence is high and increasing [3,4]. BCCs are slow growing and originate from the basal layer of the epidermis. In most cases, the tumour is limited to the epidermis and superficial layers of the dermis. However, if disregarded over time, invasive growth and local tissue destruction may occur [5]. Metastatic BCC (MBCC) is extremely rare, with estimated rates ranging between 0.003 and 0.55% [3,5]. Some histological subtypes of BCCs have a greater probability of aggressive local behaviour, more frequently have local recurrence and are more likely to metastasize [6,7]. These are defined as high-risk and are recognized as micronodular, infiltrating, sclerosing/morphoeic, basosquamous and sarcomatoid [6,7]. The spread in order of frequency is usually to the lymph nodes, lungs, bones, skin, and other sites [6,7]. Unfortunately, the prognosis for MBCC is very poor, and there is an estimated mean survival ranging from 8 months to 3.6 years [3].

In this article, we present a rare case of severe metastatic BCC to the bone marrow, three months after excision of the locally advanced tumour.

## 2. Case Report

**Figure 1:**
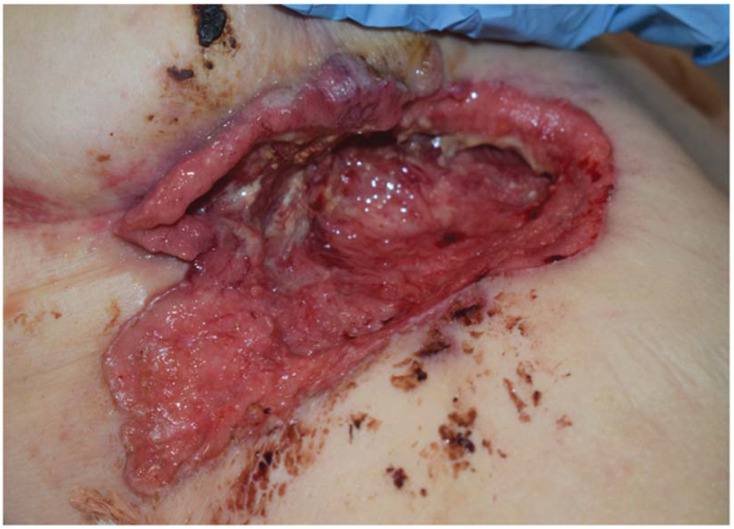
Ulcerative tumour. The picture shows a large infiltrating and ulcerative tumour inferior of the right breast.

**Figure 2:**
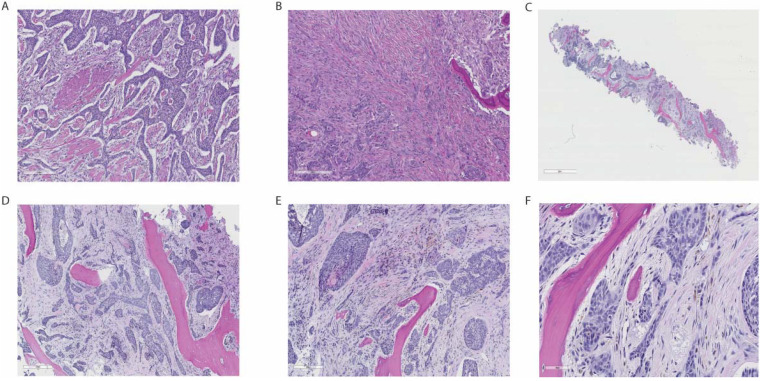
Histopathological features of metastatic BCC. The images show histopathological features from the original skin tumour (**A**,**B**), and from bone marrow biopsy with metastases (**C**–**F**). (**A**) Tumour showing irregular growth of basaloid tumour islets with palisading of nuclei and cleft formation peripherally in the tumour islets. (**B**) Strikingly infiltrative growth pattern (morphoea-like) with invasion and destruction of rib bone tissue. (**C**) Bone marrow biopsy low-power view showing extensive infiltration of marrow spaces. (**D**) Bone marrow infiltrated by malignant tumour tissue growing in irregular islands. (**E**) Irregular tumour islands with destruction of bony trabeculae. (**F**) Tumour cells are medium sized with light eosinophilic cytoplasm with round to oval nuclei with somewhat uneven nucleus contour and palisading, compatible with metastatic BCC.

The patient was a 68-year-old woman, with a medical history of paranoid schizophrenia spanning more than 10 years. Six months prior to admission, she had experienced a weight loss of more than 10 kg and was complaining of generalized pain. She was suffering from delusions and refused medical examination. During hospitalization in a psychiatric unit, a large ulcer under her right breast was discovered, measuring 20 × 10 centimetres and the ulcerating crater seemingly infiltrating the intercostal muscles and affecting the visceral pleura (Figure 1). There were no signs of infection or profuse bleeding. An ultrasonography of the breast was performed with no other reported pathology. Biopsy showed BCC with a mixed histological growth pattern, including morphoea growth in several areas. Further, the tumour showed microscopic infiltration into adjacent connective and adipose tissue. Immunohistochemistry was positive for BerEP4, cytokeratin 5/6 and focal areas positive for cytokeratin 7, whereas EMA was negative. These findings were in concordance with basal cell carcinoma. Biopsy showed infiltration of skeletal muscle, but no infiltration of the skeleton (Figure 2).

Computed tomography (CT) did not reveal any signs of metastasis, and subsequently it was decided to perform surgery with curative intention. A bloc resection was performed with the 4–7th rib including free margins of three centimetres. The operation was performed successfully, and the patient was discharged after two weeks.

**Figure 3:**
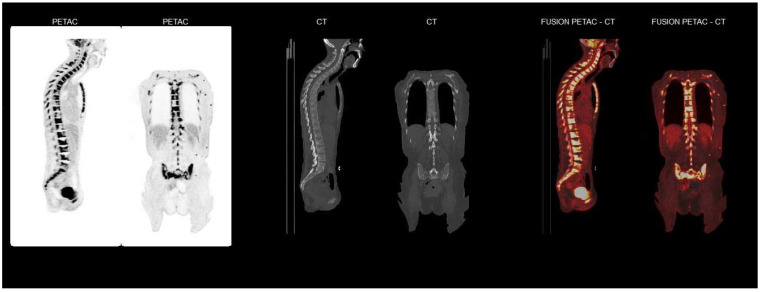
[^18^F]FDG-PET/CT of the vertebral column/bone marrow. The pictures demonstrate extensive FDG-uptake in the whole axial skeleton.

Three months post-surgery, the patient was readmitted due to gross weight loss of 20 kg, obstipation, and severe malnourishment. Blood work showed normocytic anaemia and elevated inflammation markers, as shown in Table 1. Clinical examination did not reveal any signs of local relapse. Due to increasing inflammation markers, anaemia, and a medical history suspect of occult malignant disease, fluorodeoxyglucose positron emission tomography/computed tomography ([^18^F]FDG-PET/CT) was performed. The PET-CT scan demonstrated intense FDG uptake in the entire skeleton, and extensive infiltration of the bone marrow and subcutaneous tissue (Figure 3). A bone marrow trephine biopsy taken from the iliac crest confirmed extensive BCC infiltration, with tumour cells demonstrating distinctive basaloid characteristics (Figure 2). Immunohistochemistry was positive for Ber-EP4, p40, cytokeratin 5/6c and cytokeratin 7, whereas EMA was negative. This result was in concordance with the findings from the original histopathological sections from the previous tumour operation. Histological signs indicated that the carcinoma could be classified as a basosquamous carcinoma, a variant of BCC that is known to be more aggressive [2].

Due to the extent of the patient disease and impaired general condition, no active palliative treatment with hedgehog inhibitors was offered. Palliative care was initiated, and she died only seven weeks after the diagnosis of metastatic disease.

## 3. Discussion

Even though BCC is considered to be a clinically benign skin cancer, and rarely metastasizes, risk factors such as recurrent or neglected tumours, high-risk subtypes of BCC, perineural invasion and large sized tumours exist [8]. There are few known cases where BCC metastasizes to bone marrow, as in our current patient. A recent review spanning from 1981 to 2011 identified only 172 cases of primary cutaneous BCC with histological confirmed metastasis not resulting from direct tumour spread. Among these 172 cases, 24 had spread to bone or bone marrow [9,10]; metastasis to the bone marrow is an exceptional case.

In general, the prognosis of MBCC is poor, with data suggesting a median survival around 10 months after diagnosis [3,9]. The patient we presented in this report died seven weeks after diagnosis of metastasis to the bone marrow. Tumour size and neglect of treatment are very poor prognostic markers [9], also shown in this case. Histological examination classified the carcinoma as basosquamous, a subtype of BCC known to be more aggressive and at higher risk of recurrence. The immunohistochemistry was positive for BerEP4 and negative for EMA. This in contrast to squamous cell carcinoma, which is usually positive for EMA and negative for BerEP4 [11].

The standard treatment for BCC is surgery, but non-surgical therapies have been developed for both superficial lesions and advanced BCC [1]. Patients with locally advanced and metastatic BCCs should be offered treatment with hedgehog inhibitors, vismodegib or sonidegib [12]. Studies have indicated a response, although the response is better for locally advanced disease more than metastasized BCC [1]. It is recommended that therapy for MBCC should be discussed by a multidisciplinary tumour board [12]. The recognition of MBCC is more important than ever with the development of targeted therapies such as hedgehog inhibitors [13] and cemiplimab [14].

MBCC is a rare outcome of BCC; however, it has severe consequences and poor prognosis. Therefore, it should not be overlooked. It is critical with early detection and therapy of BCC to prevent metastases, and even though we still have little knowledge to predict which BCCs are likely to metastasize [15], it can be justified that patients with multiple risk factors undergo long-term follow up for prevention [16,17].

## 4. Conclusions

MBCC is rare, but the outcome is often severe with poor prognosis. One of the most important preventive measures for metastatic outcome in BCC is early detection, treatment and in some cases, it can be beneficial with long-term follow up.

## Figures and Tables

**Table 1:** Blood tests.

Blood Tests	Values	References
Leukocytes (×10^9^/L)	8.6	3.5–11.0
Haemoglobin (g/dL)	9.4	11.7–15.3
MCV (fL)	82	82–98
Reticulocytes (×10^12^/L)	0.046	0.02–0.08
Thrombocytes (×10^9^/L)	288	165–387
CRP (mg/L)	152	<5
Procalcitonin (µg/L)	<0.10	<0.10
SR (mm)	77	<20
Creatinine (µmol/L)	41	45–90
ALP (U/L)	211	35–105
Ferritin (µg/L)	1919	15–200
LDH (U/L)	582	105–205
Albumin (g/L)	28	39–48

The table demonstrates the most relevant blood test at readmission for the patient: Abbreviations; MCV, mean corpuscular volume; CRP, C-reactive protein; SR, sedimentation rate; ALP, alkaline phosphatase; LDH, lactate dehydrogenase.

## Data Availability

The data presented in this study are available on request from the corresponding author.
